# Decoding IBS: a machine learning approach to psychological distress and gut-brain interaction

**DOI:** 10.1186/s12876-024-03355-z

**Published:** 2024-08-15

**Authors:** Astri J. Lundervold, Julie E. Billing, Birgitte Berentsen, Gülen A. Lied, Elisabeth K. Steinsvik, Trygve Hausken, Arvid Lundervold

**Affiliations:** 1 Department of Biological and Medical Psychology, Universtity of Bergen, Bergen, 5020 Norway; 2 National Center for Functional Gastrointestinal Disorders, Department of Medicine, Haukeland University Hospital, Bergen, 5021 Norway; 3 Department of Clinical Medicine, University of Bergen, Bergen, 5020 Norway; 4 Center for Nutrition, Department of Clinical Medicine, University of Bergen, Bergen, 5020 Norway; 5 Department of Biomedicine, University of Bergen, Bergen, 5020 Norway; 6 Medical-AI, Mohn Medical Imaging and Visualization Centre, Department of Radiology, Haukeland University Hospital, Bergen, 5021 Norway

**Keywords:** Irritable bowel syndrome (IBS), Fatigue, Sleep disturbances, Depression-anxiety, Cognition, Machine learning, Feature importance

## Abstract

**Purpose:**

Irritable bowel syndrome (IBS) is a diagnosis defined by gastrointestinal (GI) symptoms like abdominal pain and changes associated with defecation. The condition is classified as a disorder of the gut-brain interaction (DGBI), and patients with IBS commonly experience psychological distress. The present study focuses on this distress, defined from reports of fatigue, anxiety, depression, sleep disturbances, and performance on cognitive tests. The aim was to investigate the joint contribution of these features of psychological distress in predicting IBS versus healthy controls (HCs) and to disentangle clinically meaningful subgroups of IBS patients.

**Methods:**

IBS patients ($n = 49$) and HCs ($n = 28$) completed the Chalder Fatigue Scale (CFQ), the Hamilton Anxiety and Depression Scale (HADS), and the Bergen Insomnia Scale (BIS), and performed tests of memory function and attention from the Repeatable Battery Assessing Neuropsychological Symptoms (RBANS). An initial exploratory data analysis was followed by supervised (Random Forest) and unsupervised (K-means) classification procedures.

**Results:**

The explorative data analysis showed that the group of IBS patients obtained significantly more severe scores than HCs on all included measures, with the strongest pairwise correlation between fatigue and a quality measure of sleep disturbances. The supervised classification model correctly predicted belongings to the IBS group in 80% of the cases in a test set of unseen data. Two methods for calculating feature importance in the test set gave mental and physical fatigue and anxiety the strongest weights. An unsupervised procedure with $K = 3$ showed that one cluster contained 24% of the patients and all but two HCs. In the two other clusters, their IBS members were overall more impaired, with the following differences. One of the two clusters showed more severe cognitive problems and anxiety symptoms than the other, which experienced more severe problems related to the quality of sleep and fatigue. The three clusters were not different on a severity measure of IBS and age.

**Conclusion:**

The results showed that psychological distress is an integral component of IBS symptomatology. The study should inspire future longitudinal studies to further dissect clinical patterns of IBS to improve the assessment and personalized treatment for this and other patient groups defined as disorders of the gut-brain interaction. The project is registered at https://classic.clinicaltrials.gov/ct2/show/NCT04296552 20/05/2019.

## Introduction

Irritable bowel syndrome (IBS) is a heterogeneous gastrointestinal (GI) condition, where the patients are recognized by recurrent abdominal pain on average at least 1 day/week in the last 3 months, and altered bowel function (consistency and/or frequency) [1]. IBS affects approximately 10% of the population [2, 3], with epidemiological studies showing a somewhat higher prevalence [1] and a more frequent contact with health professionals in women than men [4]. There are different clinical subtypes related to bowel habit abnormalities [5], and the heterogeneity within the group is large, including patients with mild to moderate symptoms to individuals where the disorder has a debilitating effect on the quality of life and their general health status [4].

Despite its high prevalence and burden of symptoms, the etiology and pathophysiology of IBS are still incompletely understood [6–8]. The most well-established model of IBS refers to the bi-directional connection between the gut and the brain [9]. This has led to the definition of IBS within the umbrella term of disorders of the gut-brain interaction (DGBIs). In this model, psychological symptoms are not only defined as secondary to or co-existent with the GI symptoms but rather as an integral part of the disorder [10].

In the present study, the focus is on a selection of features that we define within the broader concept of psychological distress: fatigue, sleep disturbances, and emotional and cognition functions. Fatigue is an obvious contributor to psychological distress in patients with IBS by interfering with their ability to perform physical, mental, and social activities [11]. The prevalence of fatigue among patients with IBS is shown to be high. In a review from 2016, Han and Yang [12] reported that fatigue affected 60% of their sample of IBS patients ($n = 176$)). Such a high prevalence was supported in a more recent review paper by Shiha and Aziz [13], reporting a pooled fatigue prevalence of 54.2% in the IBS sample compared to 25-30.5% in a general population sample. They further emphasized the multidimensional impact of fatigue on daily life functioning. In contrast to Hahn and Yang [12], who suggested that fatigue could be experienced independent of other symptoms, they reported a close association between the severity of IBS symptoms and fatigue, along with associations between fatigue, anxiety, and depression.

Anxiety and depression are two symptom clusters that are not only experienced by a large group of patients with IBS but are also definitely linked to the concept of psychological distress. Recent studies have provided insights into the importance of this link, particularly in the context of the COVID-19 pandemic (see e.g. [14]). Moreover, anxiety and depression are also well-known symptom clusters among patients with IBS. A study by Midenfjord et al. [11] showed that more than 40% of a large group of patients with IBS ($n = 769$) showed a score above a clinical cutoff value for anxiety (44.9%) and between 20 and 30% on the depression subscale (25.7%) from the Hospital Anxiety and Depression Scale (HADS). The authors also confirmed a close association between anxiety/depression and fatigue in patients with IBS. They concluded that these aspects of psychological distress seem to be intertwined with the clinical and pathophysiological picture of this condition. Some studies have even indicated a common genetic basis between fatigue and anxiety-depression conditions [15, 16], and a recent classification study by Haleem et al. [17] showed that a cluster of specific symptoms of fatigue and anxiety was found to identify patients with IBS versus healthy controls at a high level of accuracy.

Sleep disturbances include another set of symptoms that are frequently reported by patients with IBS. A national US study found that 73% of patients with IBS compared to 37% of healthy controls reported sleep-related problems [18], and a systematic review by Tu et al. [19] emphasized a close relationship between sleep disturbances and severity of GI symptoms. A bidirectional association has also been reported between sleep quality and emotional symptoms [20], while few studies have investigated sleep-fatigue relationships. A study in a sample of shift workers should be of interest in this context [21]. IBS, which was found to be the most prevalent disorder of their sample, was associated with sleep disturbances, and poor sleep quality was markedly associated with emotional problems (anxiety and depression) and fatigue. Furthermore, the study showed that most participants (up to 80%) also struggled with cognitive problems affecting concentration and/or memory function. A bidirectional link between sleep problems and cognitive function [22, 23] should thus be expected in patients with IBS.

Although cognitive function has a key role in emotion regulation and all goal-directed behavior [24] there are still few studies focusing on cognitive function in patients with IBS. Cognitive function is, however, expected to be essential in a disorder where the patients are confronted with continuous streams of bothersome sensory information from the body [25]. It should also be closely related to another complaint commonly expressed by patients with IBS, namely “brain fog”, a condition characterized by forgetfulness, confusion, lack of concentration, and mental clarity [26]. Among the few available studies there are reports of impairment related to memory function, and attention/executive function [27, 28], at least in a subgroup of IBS patients [29, 30]. The relation between cognitive impairment and emotional function is well established in several diseases (see e.g., [31]), but less established in IBS. This is also true for the association between cognitive function and fatigue. Although disturbed concentration is one of the symptoms of fatigue, a low association is shown in medical conditions like post-COVID-19 [32] and a range of other disorders [33].

Fatigue, sleep disturbances, and emotional and cognitive problems, each and together, are expected to have a bidirectional association with the somatic symptoms of IBS [34]. In other words, they may be prompted by the condition, as well as lead to secondary effects on the somatic symptoms associated with IBS [35]. Fragmentation of sleep may for example be related to a need to use the bathroom during the night or abdominal pain [36], but may also intensify the GI symptoms. Emotional symptoms may be related to shame, dissatisfaction with the body, and social withdrawal associated with the GI symptoms, but emotional symptoms like anxiety and depression may also intensify or increase the frequency of abdominal pain [13]. The somatic symptoms may also be influenced by cognitive processes in patients with IBS, for example through negative cognitive biases [28], while cognition may intensify challenges associated with the disorder. A similar bidirectional relationship can be described between fatigue and GI symptoms. Furthermore, the importance of grading severity level on each of the features described above should be emphasized, as illustrated by symptoms of fatigue. Although mild symptoms, which are probably experienced in periods by all adults, should be tolerated, the psychological distress may be difficult to handle in the more severe end of a fatigue scale, where the patients are characterized by a profound lack of energy, brain fog, and disturbances of mood and sleep [37].

This complex picture of psychological distress inspired us to investigate the joint distribution and feature importance of psychological distress features using a machine learning framework. This framework has been successfully used in previous studies of IBS, both to personalize diets based on individual gut microbiome profiles [38] and to improve the accuracy of identifying IBS patients versus controls based on different features in colonic endoscopy images [39]. Others have combined GI information with psychological variables to identify more homogeneous subgroups in clinical [40] and population-based samples [41]. The study by Elahe Mousavi et al. [42] is of particular interest in the context of the present study. With a large sample of IBS patients (*n* = 988) and the inclusion of a wide range of gastrointestinal and psychological variables, they identified nine clinically relevant clusters. Interestingly, all clusters were described with different levels of psychological burden.

This motivated us to run a study focusing on psychological distress. While Haleem et al. [17] investigated items within those features, the present study defined psychological distress from symptom severity along the full scale of well-known assessment instruments. The ultimate goal was to identify patterns of psychological distress factors that should be a target in a personalized treatment procedure for this group of patients. To that end, we used a supervised procedure to classify a participant as IBS versus healthy control (HC), followed by an unsupervised data-driven procedure to investigate if we could discover meaningful clusters of IBS patients independent of the predefined labels of IBS and HC.

## Materials and methods

### Participants

The participants were part of the Bergen Brain-Gut project, which was conducted at Haukeland University Hospital, Bergen, Norway in 2020 - 2022. For details of the project design, see [43]. In this project, each patient and a group of HCs took part in an examination assessing a wide range of psychological and GI-related features. Patients with IBS and HCs were recruited through media and flyers, and some of the patients were recruited at the outpatient clinic at the hospital. All participants were at least 18 years old and were screened by a nurse according to the inclusion and exclusion criteria (see Table 1) via a phone call. They were then asked to complete a set of questionnaires, to take part in a magnetic resonance imaging (MRI) examination, and to provide stool samples. For the patients, the examination also included ultrasonography, sigmoidoscopy, upper endoscopy, and blood tests. The participants of the present study are restricted to those responding to all the measures selected for this study (see description in the Method section) and comprised $n = 49$ patients with IBS and $n = 28$ HCs. It should be noted that a large proportion of these participants were also included in a previous study [17], focusing on a more restricted part of the features and using another analytic approach than in the present study. According to Rome IV phenotype definitions, 19 patients with IBS were classified as IBS-D (diarrhea-predominant), 25 as IBS-M (mixed), and 5 as IBS-C (constipation-dominant).

**Table 1:** Exclusion and inclusion criteria for the IBS patients. Source: Retrieved from [43]

**Inclusion**:	Rome-IV criteria: Recurrent abdominal pain average at least 1 day/week during the last 3 months, and associated alterations in bowel habits at least 6 months before diagnosis. Other causes are excluded.
	Normal diet at least 3 weeks before inclusion IBS score equal to or above 175.
**Exclusion**:	Pharmacological treatment affecting GI tract, including medication for anxiety and depression, diabetes, coeliac disease, IBS, Polycystic ovary syndrome, active Helicobacter pylori infection, Parkinson’s disease, amyotrophic lateral sclerosis, or Psychiatric disorders.
	Treated with antibiotics for the last 3 months
	Diets such as vegetarian or vegan
	Use of probiotics or low-FODMAP diet within the last 3 weeks
	Previous intestinal surgery, except appendectomy
	Metallic implants, claustrophobia, incompatible with MRI
	Travel outside Europe last 3 weeks
	Plan to travel in the near future
	Pregnancy

### Measures

#### Age, gender, and severity of IBS

Age and gender were self-reported. The IBS-Severity Scoring system (IBS-SSS) was included to measure severity of IBS symptoms. The questionnaire includes five items assessing the severity of abdominal pain, distention, bowel habits, and quality of life [44]. The maximum score for each question is 100. A sum of scores $< 75$ is defined as normal, while scores in the ranges [75, 175), [175, 300], and $>300$ define mild, moderate, and severe IBS symptom levels, respectively [44]. The inclusion criteria for the IBS patients in the present study was an IBS-SSS score $\ge 175$. Almost all HCs obtained a score in the normal range, with a few reporting a score within the mild IBS-SSS level.

#### Chalder Fatigue Scale (CFQ)

The Chalder Fatigue Scale (CFQ) [45] was included as a measure of mental and physical fatigue. The participants are presented with 11 questions and are expected to report how they perceived their level of fatigue the previous days. On each item, they are asked whether they experience a given symptom as present *less than usual, not more than usual, more than usual, or much more than usual*. In the present study, those responses are scored according to a bimodal system, where the two first response categories are used to define the absence of a symptom (0 points) and the two latter as the presence of a symptom (1 point). The present study includes two subscales of fatigue: a physical (items $1 - 7$) and a mental fatigue scale (items $8 - 11$), denoted as F1 and F2 in the result section.

#### Hospital Anxiety and Depression Scale (HADS)

Hospital Anxiety and Depression Scale (HADS) [46] is a self-report questionnaire that is commonly used to screen for anxiety and depression in psychiatric and somatic care. HADS includes fourteen questions, where the participants are asked to rate if the content of the items matches their feelings the past week. They are given three response options: *not at all*, *from time to time (occasionally)*, *a lot of the time*, *most of the time*, coded from 0 to 3, with a max score = 42. A split in two factors, one as a measure of anxiety and one as depression, has been supported by several studies [47], denoted as H1 and H2 in the result section.

#### Bergen Insomnia Scale (BIS)

The Bergen Insomnia Scale (BIS) is included to measure sleep disturbances. The scale contains six questions, each ranked on a Likert scale from $0-7$, indicating how many days a week during the past month they have experienced a given symptom [48]. In the present study, we have divided the scale into two subscales: One subscale is called “sleep quantity”, including items indicating prolonged onset before sleep, long nocturnal awakenings, and early morning awakening (items $1 - 3$); and a “quality of sleep” subscale, including items assessing daytime sleepiness/tiredness, non-restorative sleep, and dissatisfaction with sleep (items $4 - 6$). These two scales will be referred to as B1 and B2 in the results section.

#### Repeatable Battery for the Assessment of Neuropsychological Status (RBANS)

All participants performed the tests included in the Norwegian A version of the Repeatable Battery for the Assessment of Neuropsychological Status (RBANS), administered and scored according to the instructions presented in the test manual [49]. The test battery includes a total score and subscores within the following cognitive domains: immediate memory, visuospatial abilities, verbal skills, attention, and recall. Two RBANS subscales (raw scores) were selected for the present study, a *memory* subscale and an *attention* subscale, to represent domains where previous studies have shown reduced performance in patients with IBS [27, 29]. The memory subscale is calculated as the sum of scores obtained on three subtests where the participant is asked to recall a list of words, a story, and a set of figures presented in the first part of the test procedure (a delay of approximately 20 minutes). The attention subscale is calculated as the sum of scores obtained on a Digit Span and a Coding test. The recall and attention subscales will be referred to as R1 and R2 in the results section.

### Data analysis

The analytic workflow consisted of the following steps.

(i) **Explorative data analysis**: For investigating and summarizing the statistical characteristics of the dataset, e.g., summary statistics, feature distributions, correlations, and corresponding visualizations, we used common Python libraries (Pandas, Seaborn) and interactive functionality in ydata_profiling (https://github.com/ydataai/ydata-profiling).
(ii) **Supervised classification**: For comprehensive and efficient statistical classification of IBS patients versus healthy controls based on their psychological distress features we used the open-source, low-code machine learning library *PyCaret* 3.0 library (https://pycaret.gitbook.io) in Python. Within the *PyCaret* library, essentially a Python wrapper around several machine learning libraries and frameworks, the *Random Forest Classification* (RF) was selected due to its high ranking on this type of tabular data and its superior performance in various classification tasks [50]. RF is an ensemble (a “forest”) of decision trees trained with the “bagging” method. The basic idea is to create multiple decision trees (in our case, n_tree=100) during training and to have them operate as a committee of “weak” classifiers to make the final decision [51]. The data was randomly split (70%/30%) into a training set and a test set. The first was used for iterative training and validation of the model. The latter was used in the evaluation of the model’s performance (according to several metrics) on unseen data, in order to assess its generalization ability. More specifically, the binary (*RF*) classifier was evaluated by computing the $2 \times 2$ confusion matrix (CM) and metrics derived from CM such as accuracy, recall (sensitivity), specificity, and MCC (the Matthews correlation coefficient). MCC is generally regarded as being one of the best measures of describing the confusion matrix of true and false positives and negatives by a single number [52–54]. The MCC ranges from $-1$ to 1, where 1 indicates a perfect prediction, 0 no better than a random prediction, and $-1$ indicates total disagreement between prediction and observation.
(iii) **Feature importance**: To evaluate feature importance in the *RF classification*, the *Gini index* was used for quantifying how much a given feature contributes to homogenizing the nodes within a decision tree, and thus measuring how the feature reduces uncertainty or “impurity” in the data. In this method, features more effective at creating pure nodes (i.e., nodes with predominantly one class) are considered more important [51]. Feature importance results using this method were also compared with those obtained with quite a different method, the *SHAP values* from cooperative game theory, where the features are the “players”. This was done to assess the consistency of the ranking of feature importance. The *SHAP values* of a feature is calculated by measuring its average marginal contribution to the model’s prediction across all possible feature combinations [55]. A *SHAP plot* is a tool for interpretability, providing a detailed representation of how each feature influences the model’s predictions.
(iv) **Unsupervised classification** of the eight psychological distress features was performed using the *K-means* clustering algorithm as implemented in the scikit-learn https://scikit-learn.org clustering module. This method systematically and iteratively partitions the data into *K* distinct clusters, where each data point (feature vector) is assigned to the cluster whose mean is closest, thereby minimizing within-cluster variance. The choice of $K = 3$ was selected heuristically (in a clinical sense) to distinguish between the clusters. The analysis was also re-run with different cluster numbers using the *elbow* and *silhouette* procedures to assess the “optimal” number of clusters. However, due to a small sample size and very few participants in some of the clusters when $K \gg 3$, we decided to present the analysis with three clusters. Following the convergent state of the clusters, each data point was assigned a cluster label (1, 2, or 3), and the characteristics of the cluster members were visualized and described. The inter-cluster variability was quantitatively assessed using Cohen’s *d* statistics, where values approximately 0.2, 0.5, and 0.8 denote *small*, *medium*, and *large* effect sizes, respectively, according to established statistical folklore [56]. The presentation of this metric facilitates the comparisons of the distinct characteristics of the clusters.

The implementation of the complete workflow will be openly available at https://github.com/arvidl/ibs-distress. This repository specifies the setup of a project-specific conda environment ‘ibs’ and comprises the cleaned input datasets in .csv format and Python code (*Jupyter notebooks*) for reproducing all tables and figures in the paper.

## Results

### Characteristics of the participants

The two groups were similar regarding age, with a mean value of 36.45 (SD 10.9) yrs. in the IBS and a mean of 35.82 (12.5) yrs. in the HC group. The number of females (F) was larger than for men (M) in both groups (F/M = 38/11 and F/M = 18/10, respectively). The IBS-SSS score was significantly higher in the IBS group (*p*<.001) than in the HC group (mean 499.07 and 35.82, respectively), as expected from the inclusion criteria of the present study.

### Explorative data analysis

Table 2 shows that the IBS group obtained much more severe scores than the HC group on all included variables. A statistically significant value was retained after Bonferroni correction for the quality of sleep subscale from BIS, the anxiety and depression subscales from HADS, and the physical, and mental fatigue scales from CFQ.

**Table 2:** Mean, standard deviation, median, and Mann-Whitney U test

	Age	IBS-SSS	B1	B2	H1	H2	F1	F2	R1	R2
IBS (*n*=49)										
mean	36.43	275.29	6.02	10.90	8.20	4.90	4.55	1.82	32.80	58.16
SD	10.97	71.68	3.69	4.96	4.15	3.12	2.33	1.41	5.33	8.39
median	35.00	258.00	6.00	11.00	8.00	4.00	5.00	2.00	35.00	59.00
HC (*n*=28)										
mean	34.93	35.82	3.82	6.14	3.71	1.75	1.07	0.29	35.86	62.39
SD	12.36	32.26	4.49	3.37	2.80	1.86	1.65	0.71	4.22	8.64
median	33.00	27.50	2.00	6.00	3.00	1.00	0.00	0.00	35.50	63.50
Mann-Whitney U test between IBS and HC:										
*p*	0.439	**	0.003	**	**	**	**	**	0.015	0.060

B1 (quantity of sleep), B2 (quality of sleep), H1 (anxiety), H2 (depression), F1 (physical fatigue), F2 (mental fatigue), R1 (recall), and R2 (attention) in the IBS and the HC group. ** denotes$p<0.001$

**Fig. 1:**
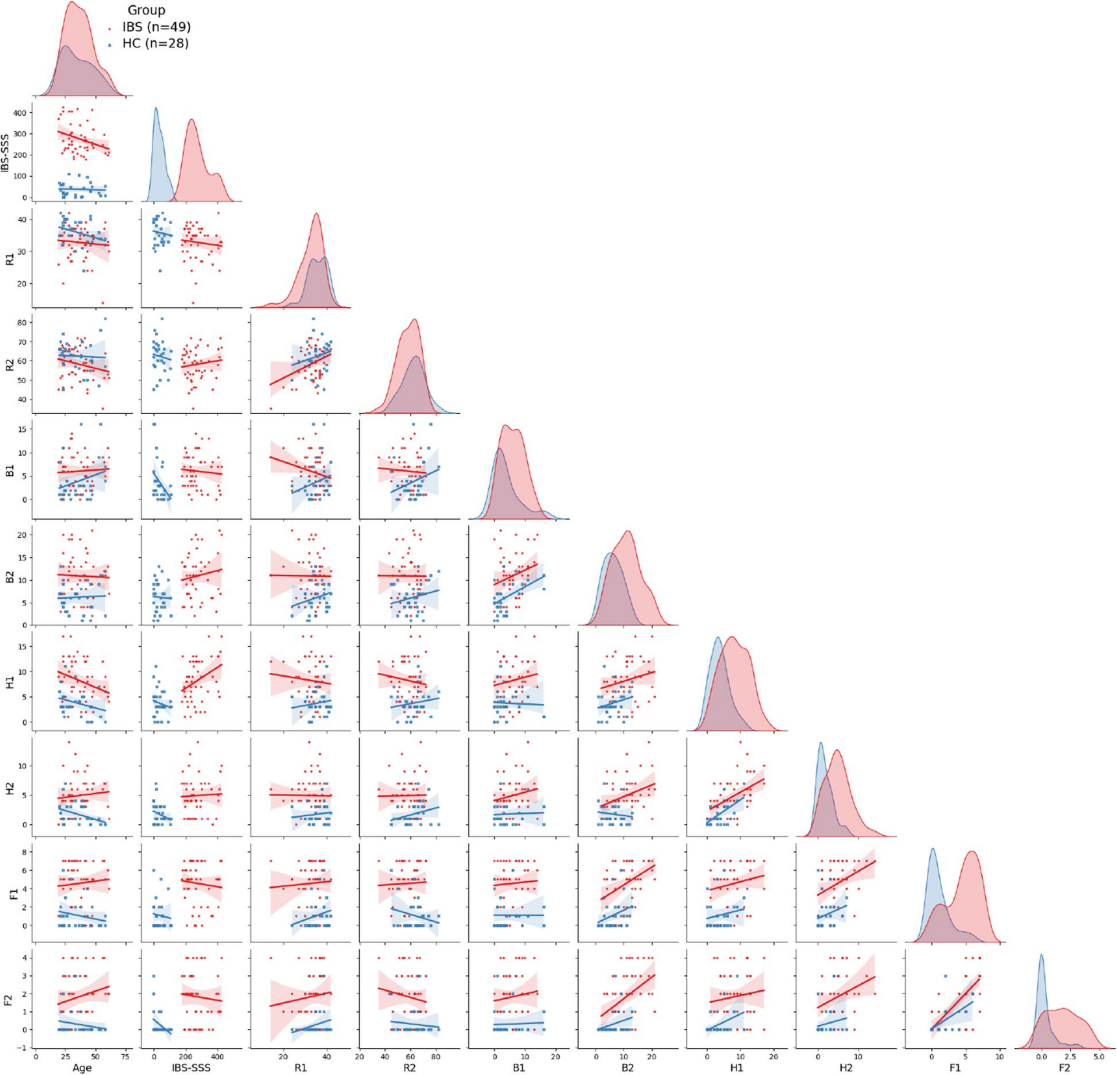
Color-coded, pairwise scatterplots of feature values observed in the IBS group and the HC group, respectively. In each panel, the IBS and the HC data points are fitted separately with a linear regression line with shaded confidence intervals. The diagonal panels show the group-wise kernel density estimation plots for the numerical features. R1 (recall), R2 (attention), B1 (quantity of sleep), B2 (quality of sleep), H1 (anxiety), H2 (depression), F1 (physical fatigue), F2 (mental fatigue). See “Measures” section for details

**Fig. 2:**
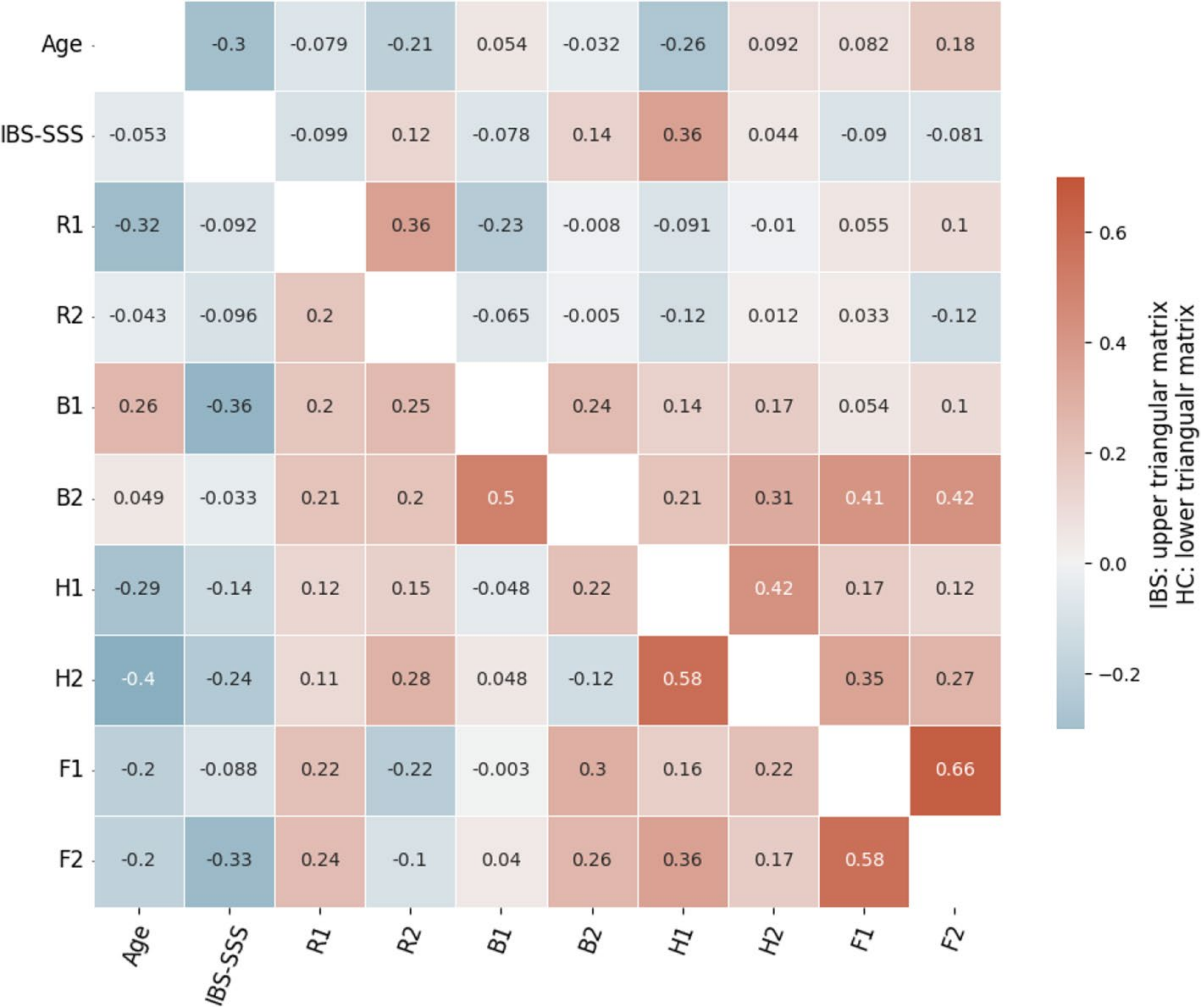
Pearson correlation matrices of pair-wise numerical features in the HC group (lower triangular) and the IBS group (upper triangular. R1 (recall), R2 (attention), B1 (quantity of sleep), B2 (quality of sleep), H1 (anxiety), H2 (depression), F1 (physical fatigue), F2 (mental fatigue). See “Measures” section for details

Figure 1 shows the color-coded, pairwise scatterplots of feature values observed in the IBS and the HC groups, respectively. The group-wise kernel density bar plots (diagonal panels) of the scores obtained by the two groups on the included subscales show that distributions of the IBS group were shifted to the right on all measures except for the two RBANS scales, where the severity follows the other direction. The heatmap in Fig. 2 shows the Pearson correlation matrices of pair-wise numerical features in the HC group (*lower* triangular) and the IBS group (*upper* triangular). In both groups, the highest pairwise correlation was found between the two CFQ subscales ($r = .58$ and .66), while the correlations between the two subscales of the HADS and the BIS were higher in the HC ($r = .58$ and .50) than in the IBS group ($r = .24$ and .42). The correlations between the two RBANS subscales were weak in both groups. In the IBS group, correlations at a moderate level were found between each of the fatigue subscales and the quality of sleep subscale from BIS ($r = .41$ and .42). All other correlations in the HC and IBS groups were below this level.

### Supervised classification

The following eight features were used in the supervised and unsupervised classification of IBS versus HC: R1 (recall), R2 (attention), B1 (sleep quantity), B2 (quality of sleep), H1 (anxiety), H2 (depression), F1 (physical fatigue), and F2 (mental fatigue).

Table 3 shows the model comparison with their metrics as obtained in the training set. Within the *PyCaret* framework, the *Random Forest Classification* was selected due to its superior performance among the tested classifiers. This classifier obtained an accuracy score above 91%, and was also high on recall, precision, and FI scores.

**Table 3:** Model comparison and their metrics. All estimators are baseline models (without hyperparameter tuning), being assessed on the *training dataset* ($n=53$, IBS: 34, HC: 19, F: 38, M: 15) with averaged stratified 10-fold cross-validation scores. We fixed the random_state=123 for reproducibility. Accuracy = $\frac{TP+TN}{TP+FN+FP+TN}$; Recall = $\frac{TP}{TP+FN}$ (sensitivity, recognition rate); Prec. = Precision $\frac{TP}{TP+FP}$ (positive predictive value); F1 = F1-score $\frac{2 TP}{2TP+FP+FN}$ (the harmonic mean of precision and recall); Kappa = Cohen’s kappa coefficient; MCC = Matthews Correlation Coefficient [53]

Model	Classifier	Accuracy	Recall	Prec.	F1	Kappa	MCC
rf	Random Forest Classifier	0.910	0.910	0.926	0.907	0.801	0.818
nb	Naive Bayes	0.893	0.893	0.920	0.889	0.774	0.798
gbc	Gradient Boosting Classifier	0.873	0.873	0.897	0.866	0.712	0.743
et	Extra Trees Classifier	0.873	0.873	0.899	0.869	0.722	0.750
lr	Logistic Regression	0.853	0.853	0.880	0.849	0.691	0.714
qda	Quadratic Discriminant Analysis	0.853	0.853	0.824	0.818		
ada	Ada Boost Classifier	0.840	0.840	0.862	0.838	0.661	0.678
lightgbm	Light Gradient Boosting Machine	0.833	0.833	0.825	0.817	0.630	0.644
ridge	Ridge Classifier	0.817	0.817	0.842	0.813	0.610	0.633
dt	Decision Tree Classifier	0.817	0.817	0.851	0.811	0.603	0.638
lda	Linear Discriminant Analysis	0.817	0.817	0.842	0.813	0.610	0.633
xgboost	Extreme Gradient Boosting	0.817	0.817	0.827	0.815	0.605	0.615
knn	K Neighbors Classifier	0.790	0.790	0.826	0.780	0.534	0.576
svm	SVM - Linear Kernel	0.757	0.757	0.690	0.699	0.353	0.377
dummy	Dummy Classifier	0.640	0.640	0.413	0.501	0.000	0.000

**Fig. 3:**
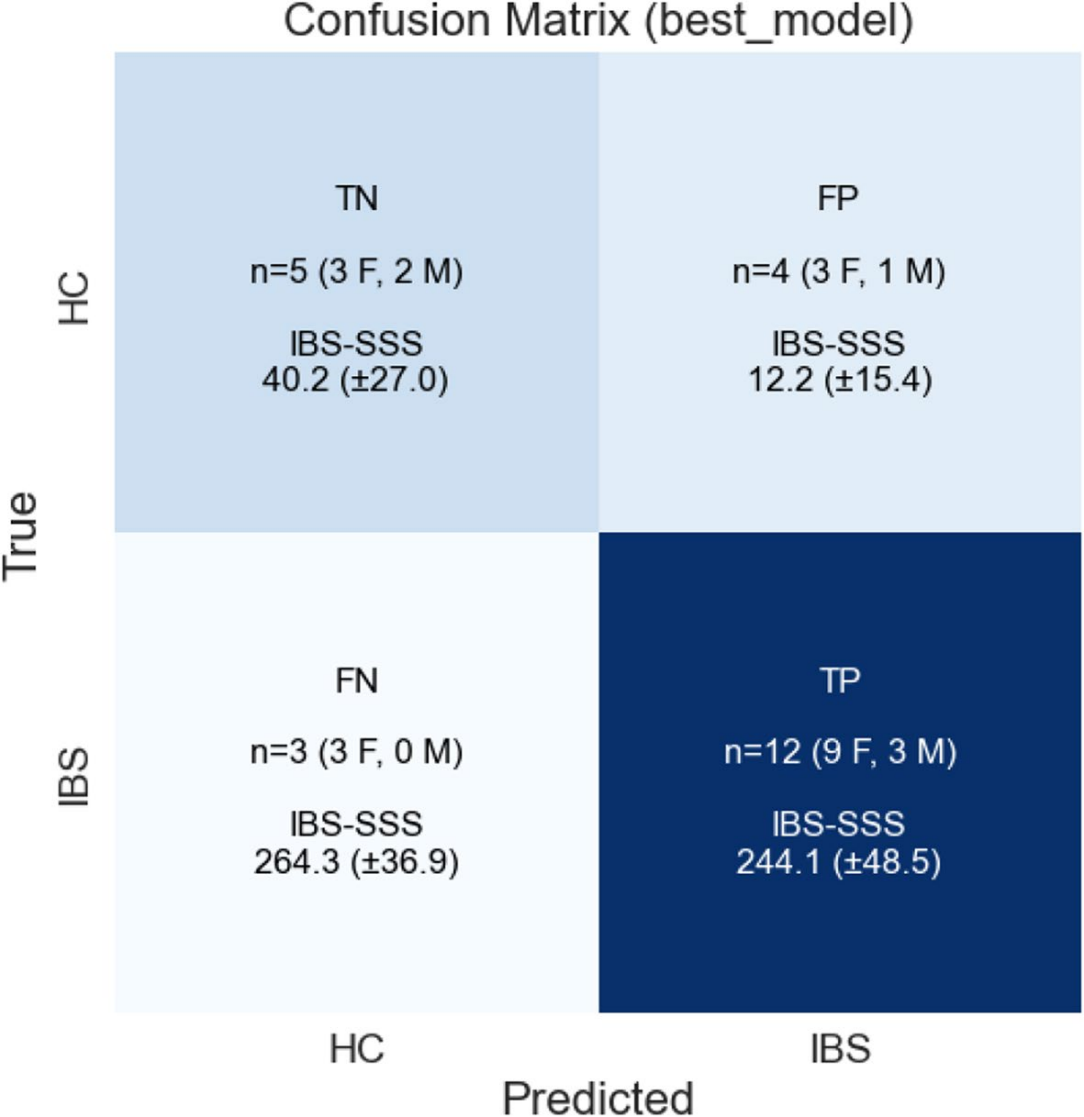
Confusion matrix, CM for the IBS and HC instances in the *test dataset* ($n=24$, IBS: 15, HC: 9, F(emales): 18, M(ales): 6). Annotation of instances (number, sex distribution, and IBS-SSS summary statistics) are depicted in each of the CM quadrants: TP (true positive), TN (true negative), FP (false positive), FN (false negative) instances predicted by the trained RF model (best_model)

Figure 3 shows the $2 \times 2$ confusion matrix for the test dataset, from which we can derive the following matrices: *Accuracy* 0.71; *Recall* 0.80; *Precision*: 0.75; *F1-score*: .77; *MCC*: .37. Thus, we find that 80% ($\frac{12}{15}$) of the IBS patients are correctly identified as IBS and that the ratio of correct classifications for the HC group ($\frac{5}{9}$) is lower (56%). An MCC of .37 suggests that our model has a moderate positive correlation between the observed and predicted classifications. In our context, this might indicate consideration of model hyperparameter tuning, or more “feature engineering” in the selection of psychological distress variables, as proposed in the Discussion. The low IBS-SSS score in the falsely HCs identified as IBS and the high IBS-SSS scores among the IBS patients falsely identified as HCs should be noted.

With these promising results, showing that the Random Forest classifier performed well on both the training set (unsurprising) and on the unseen test data, we calculated feature importance according to the two procedures described in the Methods section.

**Fig. 4:**
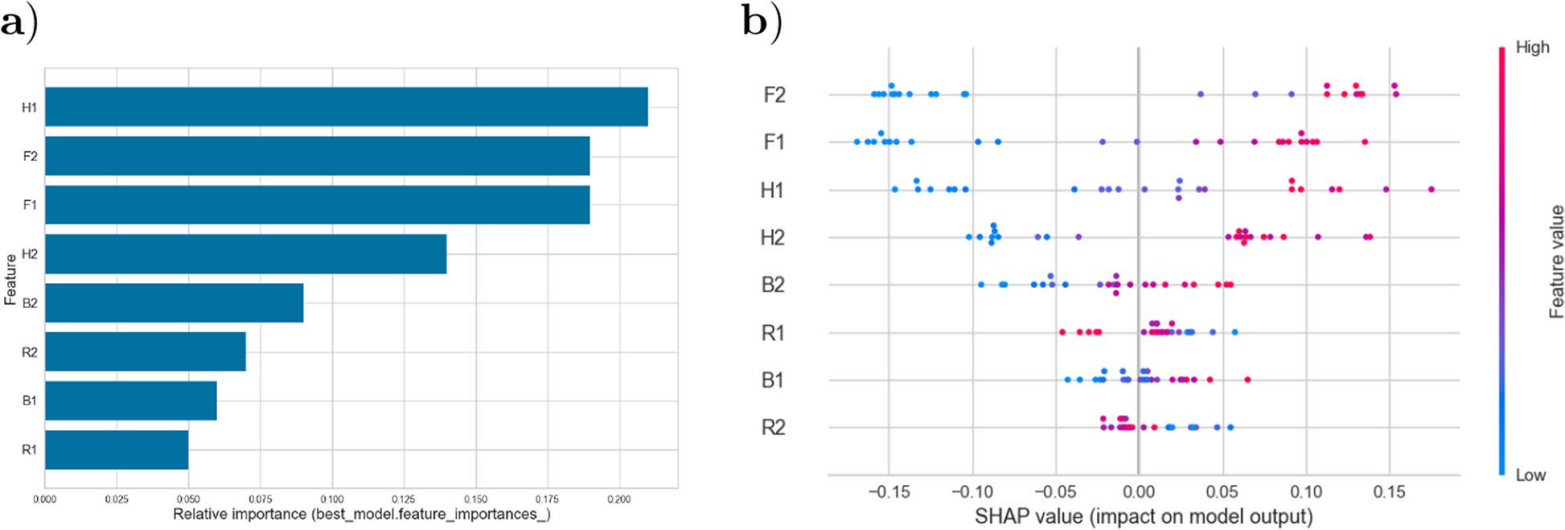
Calculation and visualization of feature importance. **a** Gini importance **b** SHAP values

Figure 4 shows that both methods identified the strongest importance of the two fatigue scales and the anxiety subscale, with a more mixed identification of features among the ones defined as those with the lowest importance. The SHAP value plot added information about the importance of the values on these measures. The y-axis in the plot reflects the impact of the feature on the model’s output and the x-axis quantifies the impact of a feature on the model’s prediction. Positive values are shown to the right of zero, with red points representing higher feature values. A high variability indicates strong diversity in the impact of the feature on the model output. Mental (F2) followed by physical fatigue (F1) are highly influential with values varying significantly across the dataset. The spread is moderate for the anxiety (H1), depression (H2), and quality of sleep (B2) subscales. The narrow spread of the other features (R1, R2, and B1) suggests they are less influential on the model output.

### Unsupervised classification

**Fig. 5:**
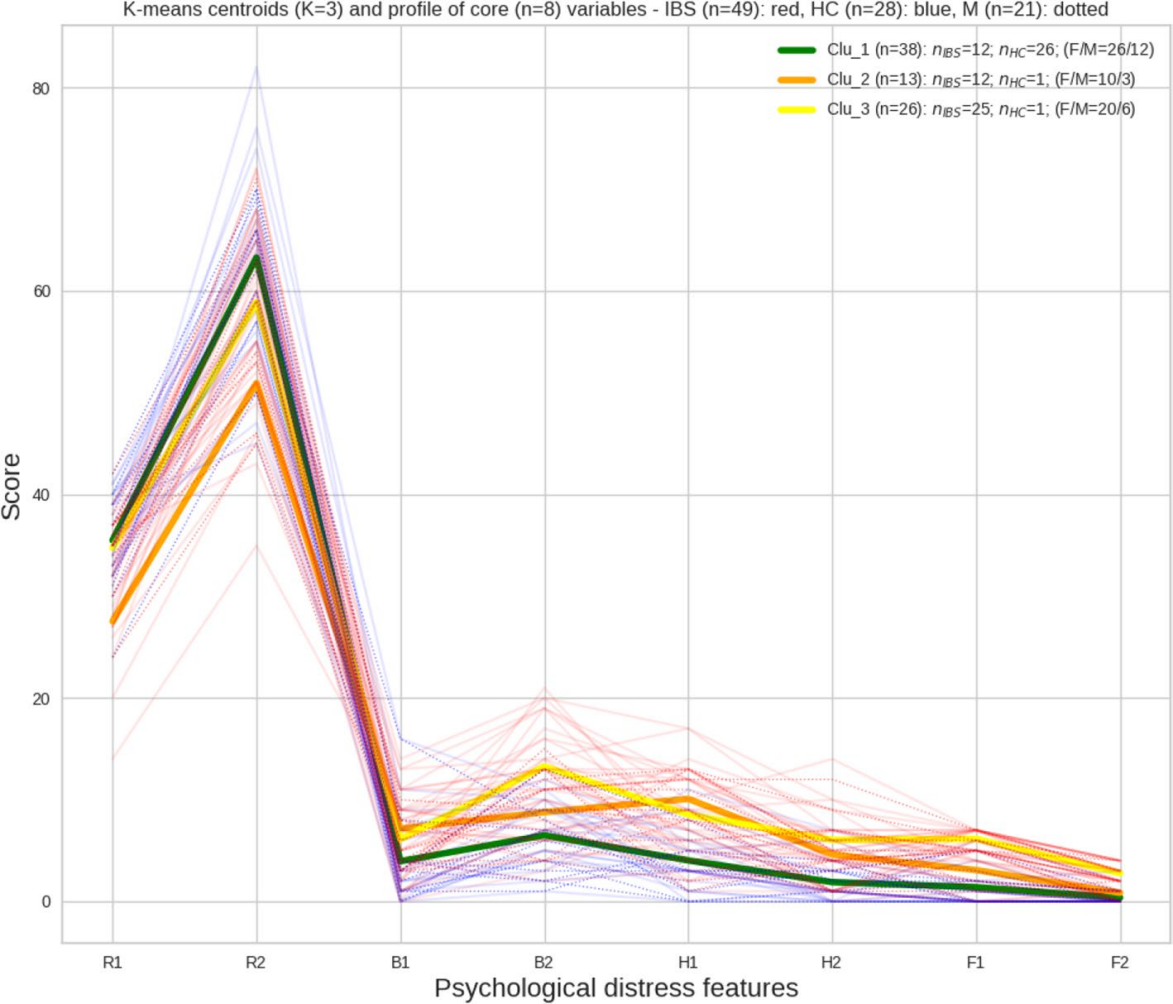
K-means clustering ($K = 3$). Line plots (thin lines) of the eight psychological distress features where the three color-coded cluster centroids (fat lines) are superimposed. HCs are colored in blue (dotted lines) and IBS in red

The K-means clustering procedure showed that all except two of the HCs were included in Cluster 1, with the largest subgroup of IBS patients (51%) allocated to Cluster 3. The higher number of males included in Cluster 3 versus Cluster 2 should be noted (Fig. 5).

Table 4 shows the means and standard deviations (SD) of the features within each cluster, with values re-scaled to their original scales, and the effect sizes of the differences between the clusters on each of their components. Overall, the scores of Cluster 1 were lower on the anxiety (H1), depression (H2), sleep disturbances (B1 and B2), and fatigue (F1 and F2) scales, and higher on the two cognitive measures of recall (R1) and attention (R2) than the two other clusters. Furthermore, the low scores on the cognitive scales in Cluster 2 and the high fatigue scales (F1 and F2) in Cluster 3 should be noted, along with the opposite directions of effects on the anxiety (H1) and depression (H2) subscales. To sum up, this indicates that while one group of IBS patients show no signs of psychological distress (Cluster 1), another cluster primarily shows scores in the most severe end on the cognitive and anxiety scales (Cluster 2), while the third cluster mainly shows the most severe problems related to sleep quality, depression, and fatigue. A follow-up analysis showed that the IBS patients allocated to the three groups were similar regarding age (mean 32.9; 32.8 and 37.4, respectively) and the IBS-SSS score (279.8; 277.1 and 272.2, respectively).

**Table 4:** The mean and standard deviations (SD), with Cohens’s d for each the components (R1, ..., F2) of the three clusters

	R1	R2	B1	B2	H1	H2	F1	F2
*Cluster 1* $n = 38$								
mean	35.50	63.26	3.95	6.50	4.03	1.89	1.39	0.37
SD	3.60	7.28	4.21	3.30	2.98	1.75	1.84	0.71
*Cluster 2* $n = 13$								
mean	27.54	50.92	7.15	8.77	10.08	4.62	3.08	0.85
SD	6.35	7.38	3.46	4.11	3.68	2.47	2.06	1.21
*Cluster 3* $n = 26$								
mean	34.77	58.88	6.12	13.27	8.54	6.04	6.15	2.77
SD	4.03	7.99	3.76	4.79	4.00	3.32	0.97	0.95
Effect size (Cohen’s d)								
*Cluster 1 vs Cluster 2*	1.80	1.69	-0.79	-0.65	-1.91	-1.39	-0.89	-0.55
*Cluster 1 vs. Cluster 3*	0.19	0.58	-0.54	1.71	-1.32	-1.66	-3.08	-2.94
*Cluster 2 vs. Cluster 3*	-1.47	-1.02	0.28	-0.98	0.39	-0.46	-2.17	-1.84

R1 (recall), R2 (attention), B1 (quantity of sleep), B2 (quality of sleep), H1 (anxiety), H2 (depression), F1 (physical fatigue), and F2 (mental fatigue)

## Discussion

### Summary of the results

The study explored the presence and severity of psychological distress among patients with IBS. Overall, our findings align with prior research, indicating significantly more severe fatigue [13], anxiety and depression [11], sleep disturbances [18], and reduced performance on psychometric tests of attention [27, 28] and memory function [29] in patients with IBS compared to a group of healthy controls (HCs). Pairwise correlation analyses revealed the strongest relationship between fatigue and quality of sleep measures (for details, see Table 2 and Fig. 2). The application of a Random Forest Classification model, which successfully classified 80% of the IBS patients in a hold-out test dataset, and accompanying analysis of feature importance, pinpointed mental and physical fatigue (F2 and F1), along with anxiety (H1), as the most important features for discriminating between IBS patients and HCs. Variability in the impact of the different psychological distress variables in the predictive *RF* model, as revealed through *SHAP value* analysis, suggested a substantial diversity of psychological distress among IBS patients. A fully data-driven, unsupervised *K-means* clustering approach supported this diversity by identifying three distinct subgroups of IBS patients. One cluster appeared largely unaffected by psychological distress, another exhibited low performance on the cognitive tests and elevated symptoms of anxiety, and the third was characterized by the most severe fatigue, sleep quality disturbance, and symptoms of depression. Notably, the severity of IBS symptoms (IBS-SSS) and age range among the IBS patients were similar across the clusters. However, the cluster allocation of male participants was uneven across the subgroups, suggesting a potential gender-related pattern in the experience of psychological distress among IBS patients.

### Fatigue and its relation to sleep disturbances

In our investigation of psychological distress, particular attention was given to the role of fatigue - a symptom that is known to profoundly influence both our physical and mental well-being [13]. Through the application of our machine learning approach, enabling the concurrent analysis of various factors, we identified physical and mental fatigue as critical elements in distinguishing between the experience of IBS patients and HCs in the supervised classification. This finding was supported by two methods used to assess feature importance (*Gini impurity* and *SHAP values*). Moreover, the differentiation in severity level of fatigue across the three clusters in the unsupervised classification supported the notion that fatigue should be considered along a continuum from a mild to a severe level in patients with IBS [12, 57].

Previous research has shown that fatigue, along with other features of psychological distress, may have a substantial impact on quality of life. This was emphasized in a systematic review by Ohlsson [58]. Interestingly, our study revealed weaker pairwise correlations between fatigue and the other features of psychological distress than expected from the many items related to sleep, emotional, and cognitive features included in the questionnaire used to assess fatigue in the present study (CFQ-11) [45]. The strongest correlation was found between the quality of sleep subscale and the fatigue scales. Previous studies have shown a close relationship between quality disturbances of sleep and sleep disturbances referred to as sleep quantity in the present study (see e.g., [19]. Here, we found a much stronger correlation between those different aspects of sleep disturbances in the HC than in the IBS group. This points to a unique pattern within the IBS group, emphasizing the importance of quality of sleep in patients with IBS and its relation to fatigue (see [22]).

### Fatigue, emotional and cognitive function

Recent research, including the study by Gilley et al. [22], has highlighted the intricate association between quality of sleep and fatigue, as well as between these features and emotional symptoms like anxiety and depression. While direct pairwise comparisons in our study showed weaker correlations than expected, the multivariate analyses revealed a more complex pattern among these features of psychological distress. Particularly, anxiety, closely followed by depression, emerged as a significant factor in our supervised classification analysis, emphasizing the critical role of emotional function in the IBS symptom complex [11]. Furthermore, our findings from unsupervised classification suggested a more nuanced picture, where anxiety and depression influence subgroups of IBS patients in distinct ways. In the two most severely impaired subgroups of IBS patients (Cluster 2 and 3), one is predominantly affected by anxiety and another by depression. This differentiation suggests a diversity in emotional symptoms within the IBS population and signals the need for more detailed investigations into how these differences contribute to the disorder.

The results regarding cognitive function should be noted. Given its key role in emotion regulation and all goal-directed behavior [24], stronger importance of cognitive measures should have been expected in a group of patients characterized by continuously bothersome sensory information from the body [25]. With the unsupervised classification analysis, however, the study contributed to a more nuanced picture by showing that performance on cognitive tests was particularly low in a subgroup of IBS patients. This finding should be underscored, as it aligns with results from previous studies from our research group. In those studies, impairment on a psychometric test of cognitive function [29] and on a self-reported questionnaire of executive functioning [30] were restricted to a subgroup of patients. These findings should thus inspire further studies of cognitive function and its interplay with emotion and physical symptoms in patients with IBS.

### The role of gender and age in IBS

Several studies have noted more severe symptoms associated with IBS in women than men [58], a gender imbalance that was reflected in the present study. This has been explained by a tendency for women to be more prone to anxiety and depression than males [59], while others have put weight on hormonal factors [60] and sociocultural influences [4]. In the present study, male patients with IBS were primarily identified in the largely unaffected group. Among the two most severity-affected clusters, however, they were more frequently allocated to the one with the most severe symptoms of fatigue, sleep disturbance, and depression. One might speculate if this supports previous studies indicating that males experience IBS symptoms differently from females [61] and that at least a subgroup of males do not seek medical attention until their symptoms are at a high severity level.

The narrow age range and the small sample size prevented age-specific analysis in the present study. Other studies have, however, shown an inverse relationship between GI symptoms and age [62], and that age may be a critical factor in understanding the pattern of psychological distress and its consequences in patients with IBS and other related disorders [63]. Taken together, these findings further underscore the *heterogeneity* of IBS, and that there may be a need for gender- and age-tailored treatment approaches.

### Clinical implications

A key finding of the present study was the identification of distinct, clinically valid subgroups, each with different levels of symptom severity and cognitive functioning. This insight should be critical for healthcare providers to consider when creating personalized treatment plans. Recent reports from the Rome working team on gut-brain behavior therapies [64] have outlined various treatment programs for patients with IBS, emphasizing the effectiveness of psychological interventions, such as cognitive behavioral therapy (CBT) [65], even when administrated purely online [66]. Our study indicates that some patients, the ones in the cluster with cognitive difficulties and anxiety, may benefit from a targeted cognitive-emotional pre-treatment procedure before deciding on a treatment strategy for a given patient [67]. Although some treatment strategies may be an exception, most treatment programs presented by Keefer et al. [64] put a strong burden on cognitive and emotional control. Particularly, patients with reduced cognitive control combined with a high level of health-related anxiety may face specific challenges that must be accounted for [68]. In summary, our findings underscore the importance of screening for different facets of psychological distress in IBS patients, moving away from a one-size-fits-all strategy towards a personalized medicine approach when assessing and treating patients with IBS. In order to make the findings even more clinically relevant, future studies could use the present machine learning approach to classify IBS among a larger set of disorders of the gut-brain interaction.

### Strengths and limitations

A major strength of the present study lies in using multivariate analyses and a machine learning approach to uncover natural and potentially hidden patterns within a clinical and demographic data set. By this, we may have contributed to alertness on the heterogeneity and complexity of symptoms associated with IBS. Random Forest, an ensemble learning model, excels in handling nonlinear relationships and identifying important features without being too sensitive to noise or imbalances characterizing the data set. Additionally, by splitting our data into a training set and a separate test dataset, the results can be generalized, and the risk of overfitting can be reduced. Moreover, applying sophisticated feature importance methods, such as SHAP values with the prediction model, helps reveal the most clinically relevant factors in psychological distress related to IBS.

Despite these methodological strengths, we acknowledge several limitations. The small dataset size, including primarily females, is the most notable constraint, restricting the generalizability of the findings. This limitation also prevents more detailed clinical stratification and analysis, e.g., dividing the sample into age groups, and subgroups like IBS-C, IBS-D, and IBS-M. Furthermore, a larger set and wider source of data (e.g., imaging, physiological measurements, and microbiome sequencing) will be needed to fully explore the bidirectional communication within the gut-brain axis. We are also aware that the strict Rome-IV diagnostic criteria for IBS may have led to the inclusion of patients with higher levels of psychological distress than in previous studies, and that information about the duration of the disorder could have improved the strength of the study. The restricted ranges of IBS-SSS scores qualifying to be enrolled as IBS or HC in the study could also be relaxed, enabling a study of IBS along a continuum and using machine learning regression methods in addition to (multi-)class predictive models. Lastly, the cross-sectional design prevents the ability to conclude about disease trajectories and the dynamics of IBS, which will require a longitudinal study, preferably with interventions.

## Conclusions

In conclusion, this study shows the complex and intertwined nature of psychological distress in patients with IBS. Within a model of the brain-gut axis, the present study highlights the significant role of associations between and the joint contribution of fatigue, sleep disturbances, anxiety and depression, and performance on tests of recall and attention. Taken together, these findings support that all features are integral components of IBS symptomatology. The results also suggest different patterns of psychological distress among patients with IBS, leaving one subgroup unaffected. Although restricted by a low sample size, the study should inspire future longitudinal research to further characterize clinical patterns of IBS, and by this contribute to improving the quality of life for patients with IBS and potentially extend findings to other disorders of gut-brain interaction like chronic fatigue syndrome/myalgic encephalomyelitis (CFS/ME) and long COVID.

## Data Availability

The implementation of the complete workflow, the setup of the corresponding conda environment, the cleaned input dataset in .csv format, and code for all resulting tables and figures are available as *Jupyter notebooks* at https://github.com/arvidl/ibs-distress.

## Abbreviations

HC: Healthy controls
IBS: Irritable bowel syndrome
GI: Gastrointestinal
DGBI: Disorders of the gut-brain interaction
CFQ-11: Chalder Fatigue Scale - 11 items
BIS: Bergen Insomnia Scale
HADS: Hamilton Anxiety and Depression Scale
RBANS: Repeatable Battery for the Assessment of Neuropsychological Status
IBS-SSS: IBS Severity Scoring System
CM: Confusion matrix
MCC: Matthews correlation coefficient
RF classification: Random Forest Classification
R1: Recall
R2: Attention
B1: Quantity of sleep
B2: Quality of sleep
H1: Anxiety
H2: Depression
F1: Physical fatigue
F2: Mental fatigue
CSF/ME: Chonic fatigue syndrome/Myalgic encephalomyelitis
COVID-19: Coronavirus disease caused by the SARS-CoV-2 virus

## Data availability

Data are provided as supplementary information in the following GitHub repository: https://github.com/arvidl/ibs-distress (available if the manuscript is accepted for publication or requested by reviewers).
